# Motor Reproduction of Time Interval Depends on Internal Temporal Cues in the Brain: Sensorimotor Imagery in Rhythm

**DOI:** 10.3389/fpsyg.2018.01873

**Published:** 2018-10-02

**Authors:** Tatsuya Daikoku, Yuji Takahashi, Nagayoshi Tarumoto, Hideki Yasuda

**Affiliations:** ^1^Department of Neuropsychology, Max Planck Institute for Human Cognitive and Brain Sciences, Leipzig, Germany; ^2^Faculty of Health Care and Medical Sports, Teikyo Heisei University, Chiba, Japan; ^3^Faculty of Human Care, Teikyo Heisei University, Tokyo, Japan

**Keywords:** reproduction, temporal, time interval, rhythm, motor, auditory

## Abstract

How the human brain perceives time intervals is a fascinating topic that has been explored in many fields of study. This study examined how time intervals are replicated in three conditions: with no internalized cue (PT), with an internalized cue without a beat (AS), and with an internalized cue with a beat (RS). In PT, participants accurately reproduced the time intervals up to approximately 3 s. Over 3 s, however, the reproduction errors became increasingly negative. In RS, longer presentations of over 5.6 s and 13 beats induced accurate time intervals in reproductions. This suggests longer exposure to beat presentation leads to stable internalization and efficiency in the sensorimotor processing of perception and reproduction. In AS, up to approximately 3 s, the results were similar to those of RS whereas over 3 s, the results shifted and became similar to those of PT. The time intervals between the first two stimuli indicate that the strategies of time-interval reproduction in AS may shift from RS to PT. Neural basis underlying the reproduction of time intervals without a beat may depend on length of time interval between adjacent stimuli in sequences.

## Introduction

### Temporal Processing in the Brain

“Time” is a ubiquitous phenomenon that is observed around the world. It is a concept full of wonders, in which there remains much to be discovered. The question of how the human brain perceives time has been a fascinating topic in many fields of study. It is currently believed that there is no specific receptor in our brain for what we refer to as “time” (Poppel, 1997, 2009). Nevertheless, the perception of time is constantly required for the everyday decisions we make, and for recognition of intervals and motion as well as auditory structured information such as speech and music (Wittmann, 2009). Neurophysiological studies have revealed that there exist neural areas that contribute to the perception of time intervals although the temporal function is not the primary process of those brain regions (Matell and Meck, 2000; Kagerer et al., 2002; Mauk and Buonomano, 2004; Buhusi and Meck, 2005; Mita et al., 2009; Hironaga et al., 2017). Previous studies have proposed hierarchical neural models of temporal processing based on distinct time scales. One is an automatic system that contributes to the perception of sub-second time intervals, which recruits motor systems in the brain (e.g., the supplementary motor area and the cerebellum) (Sakai et al., 1999; Schubotz and von Cramon, 2001; Coull et al., 2004; Pastor et al., 2004) even without a motor task (Grahn and Brett, 2007; Chen et al., 2008). Another is a system that depends on attention and working memory, and contributes to the perception of supra-second time intervals, which are more connected to the right prefrontal and parietal cortical areas (Madison, 2001; Lewis and Miall, 2003; Miyake et al., 2004). Yet another is a system that contributes to the perception of time intervals of seconds to minutes, which mainly involve the corticostriatal circuits involved in the basal ganglia (Buhusi and Meck, 2005). Thus, several areas in the brain contribute to temporal processing, and its function could be vulnerable in patients with impairments in brain areas that are important for temporal processing (Kagerer et al., 2002), for example, persons with motor and other impairments such as stuttering (Toyomura et al., 2015), Parkinson’s disease, stroke (Thaut et al., 2015), dyslexia (Przybylski et al., 2013), and autism (Szelag et al., 2004b).

### Time Scale of Time-Interval Perception and Motor Reproduction

Temporal integration and disintegration is an essential system involved in time perception. For instance, the temporal order of two stimuli can be recognized if the time interval is at least 20 to 60 ms (Exner, 1875; Hirsh and Sherrick, 1961; Kanabus et al., 2002; Fink et al., 2006). That is to say, the time interval of 20 to 60 ms is a threshold of temporal disintegration of events. In contrast, at a different perceptive level, temporally adjacent stimuli between which the time interval is over 60 ms can be united into one percept. In speech perception, if the time interval between the onset of lip movement and auditory speech stimulus does not exceed 200 to 250 ms, the visual and auditory inputs can be integrated as a syllable unit (van Wassenhove et al., 2005, 2007). This suggests that the time interval of 200 to 250 ms is the specific threshold of temporal integration in multisensory speech processing. Sensorimotor processing also has a threshold of temporal integration on distinct time scales: 250 ms (Peters, 1989; Mates et al., 1994; Wittmann et al., 2001) and 2 to 3 s (Fraisse, 1984; Szelag et al., 1996; Wittmann and Poppel, 2000; Ulbrich et al., 2007; Noulhiane et al., 2008). According to studies on sensorimotor processing, up to approximately 3 s, we can accurately reproduce time intervals with small temporal variance whereas over 3 s, the time intervals of reproduction gradually become shorter (Poppel, 1971; Fraisse, 1984; Szelag et al., 1996; Wittmann and Poppel, 2000; Ulbrich et al., 2007; Noulhiane et al., 2008). These findings are also supported by neurophysiological research (Elbert et al., 1991). The event-related potentials (ERPs) were recorded when participants reproduced visual stimuli ranging in duration from 1 to 8 s. When accurately reproduced up to 3 s, slow negative shifts in the ERPs were detected. In contrast, when durations exceeded 3 s and the reproduction was becoming inaccurate, this shift was reduced. These results indicate that shorter time intervals up to 3 s can be united into one percept in working memory whereas longer time intervals over 3 s temporally disintegrate (Poppel, 1997).

### Time-Interval Perception With Internalized Cues in the Brain

Temporal integration can also occur in stimulus sequences with a beat: temporal integration consisting of beats (Szelag et al., 1998). The results, however, differ from temporal integration with no event (Szelag et al., 1996, 1997; Szelag, 1997). A higher frequency of beats leads to shorter time intervals in temporal integration whereas a lower frequency of beats leads to longer time intervals, and the longest time interval in temporal integration is approximately 3 s. Szelag and colleagues reported that a lack of events within a time interval, which leads to a low level of mental and behavioral activities, gives an impression of a slow passage of time, subjectively experienced as boredom. In contrast, many events within a time interval, which leads to a high level of mental and behavioral activities, give an impression of a quick passage of time.

According to a previous study, humans prefer to maintain a steady beat when tapping on beat with a steady sequence (Launay et al., 2014). They tend not to maintain a beat when tapping offbeat with an unsteady sequence, however. Furthermore, the strategies of tapping are influenced by the preceding time interval of stimuli. This suggests that an internal timekeeper is affected by external information on time intervals regardless of a beat (Launay et al., 2014). Thus, Poppel claimed that temporal perception depends on subjective phenomena, such as simultaneity, successiveness, temporal order, and steadiness, which exist in the human brain as internalized cues (Poppel, 1997, 2009). To the best of our knowledge, however, few studies have investigated how internalized cues with a beat and those with no beat interact with each other, and how simple perceptions of time intervals without any internalized cues differ from those with cues. Considering these previous findings, we hypothesized that temporal perception depends on the subjective conditions of internalized cues. The present study investigated how temporal processing differed among three conditions: (1) with no internalized cue, (2) with an internalized cue without a beat, and (3) with an internalized cue with a beat. To understand how the human brain perceives time, it is important to investigate how internalized temporal cues modulate time-interval perception and reproduction.

### The Purpose of the Present Study

The present study aimed to reveal how the motor reproduction of time intervals was modulated by each of three conditions: [1] with no internalized cue, [2] with an internalized cue without a beat, and [3] with an internalized cue with a beat, and further verified the relationships among the three conditions. Previous studies suggest that reproduction errors gradually become more negative as duration increases (Poppel, 1971; Fraisse, 1984; Szelag et al., 1996; Wittmann and Poppel, 2000; Ulbrich et al., 2007; Noulhiane et al., 2008). On the other hand, some studies also suggest that temporal perception depends on subjective phenomena, such as temporal order and steadiness, which exist in the human brain as internalized cues (Poppel, 1997, 2009; Launay et al., 2014). Considering these previous findings, we hypothesized that temporal perception depends on the subjective conditions of internalized cues.

**FIGURE 1:**
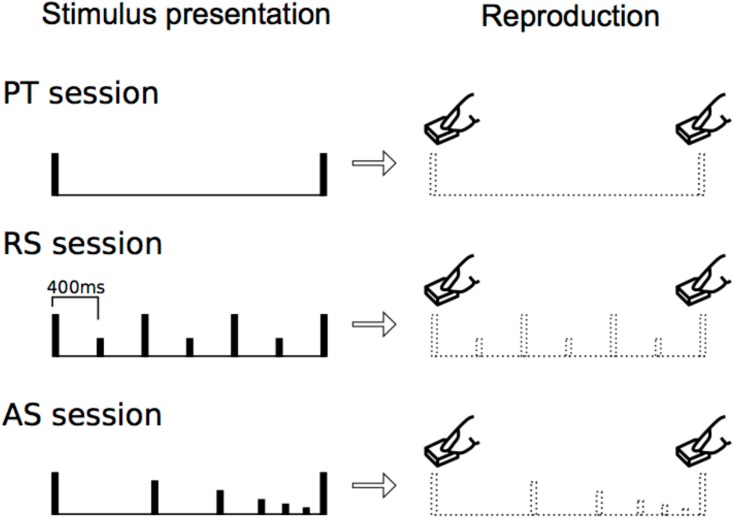
Experimental procedure. In each trial, stimulus presentation was followed by reproduction. The participants reproduced the time interval between the first and the last stimuli that were presented most recently.

In experiment 1 (paired tone session: PT), participants were presented with two stimuli (**Figure 1**, top). In experiment 2 (accelerando series session: AS), they were presented with a stimulus series in which the tempo picked up gradually (i.e., accelerando) (**Figure 1**, middle). In experiment 3 (rhythm series session: RS), they were presented with a stimulus series in which every second tone was accented (400 ms per beat) (**Figure 1**, bottom). All of the sessions in each experiment were categorized into eight types of trials with different time intervals: 0.8, 1.6, 2.4, 3.2, 4.0, 4.8, 5.6, and 6.4 s trials. They reproduced the time intervals between the first and the last stimuli by pressing a button. First, to understand how the motor reproduction of time intervals was modulated by internalized temporal cues, we performed correlation analyses among experiments and one sample *t*-test for each time interval trial in each experiment. Then, we also performed a 3 (session: PT, AS, and RS) × 8 (time: 0.8, 1.6, 2.4, 3.2, 4.0, 4.8, 5.6, and 6.4 s trials) repeated-measures analysis of variance (ANOVA). We hypothesized that, in PT sessions, the time intervals of reproduction would gradually shortened compared to those of the stimulus presentation from the time intervals of approximately 3 s, consistent with the results of previous studies by Poppel (1971, 1997). In addition, we hypothesized that internalized temporal cues of a beat and no beat differently modulate temporal processing of intervals, and that longer exposure to beat presentation leads to stable internalization and efficiency of reproduction (Launay et al., 2014), compared to exposure to no-beat presentation. The present study is the first to compare temporal processing of intervals by testing the three specific formats of internalized cues utilized here.

## Materials and Methods

### Participants

Twenty-four Japanese (10 females, mean age = 21 ± 0.2) with no history of neurological or audiological disorders were included in the data analyses. This study was approved by the Ethics Committee of Teikyo Heisei University. All participants were informed of the purpose and safety of the study, and about the protection of personal data in this experiment, and they provided written informed consent for this study.

### Experimental Protocol

The participants performed three experiments in the same order. The three experiments have forty sessions each. In each session, they completed a behavioral test in which they were presented pure tone stimuli with comfortable intensity in each participant (*f* = 440 Hz, duration = 60 ms with rise/fall of 10/10 ms, respectively). Next, they reproduced the time intervals (i.e., inter-onset interval) between the first and the last stimuli by pressing a button without sounds. The forty sessions in each experiment were randomly distributed, but could be categorized into eight types of five trials with different time intervals: 0.8, 1.6, 2.4, 3.2, 4.0, 4.8, 5.6, and 6.4 s trials.

In experiment 1 (paired tone session: PT), participants were presented with two stimuli. They then reproduced the time intervals between the two stimuli by pressing a button (**Figure 1**, top). In experiment 2 (accelerando series session: AS), participants were presented with a stimulus series in which the intensities and tempo gradually decreased and picked up (**Figure 1**). The numbers of stimuli are 3, 5, 7, 9, 11, 13, 15, and 17 in the 0.8, 1.6, 2.4, 3.2, 4.0, 4.8, 5.6, and 6.4 s trials, respectively. The intensities of the first and last stimuli are same, but those of the others decreased [dB(relative) = -3/4^∗^(a number of stimuli) + 3/4]. The tempo non-linearly picked up [0.8 s: *y* = 736^∗^ln(x) + 16, 1.6 s: *y* = 981^∗^ln(x) + 46, 2.4 s: *y* = 1200^∗^ln(x) + 58, 3.2 s: *y* = 1411^∗^ln(x) + 49, 4.0 s: *y* = 1616^∗^ln(x) + 19, 4.8 s: *y* = 1819^∗^ln(x)-28, 5.6 s: *y* = 2019^∗^ln(x)-93, 6.4 s: *y* = 2217^∗^ln(x)-17; *x* = a number of stimuli, *y* = onset time(ms)] (i.e., accelerando: **Figure 1**, middle). Participants then reproduced the time intervals between the first and the last stimuli by pressing a button. In experiment 3 (rhythm series session: RS), participants were presented a stimulus series in which every second tone was accented [400 ms per beat, or Stimulus onset asynchrony (SOA) = 400 ms] (**Figure 1**, bottom). The 0.8, 1.6, 2.4, 3.2, 4.0, 4.8, 5.6, and 6.4 s trials had 3, 5, 7, 9, 11, 13, 15, and 17 stimuli, respectively. Thus, the number of stimuli in all types of trials were the same between AS and RS sessions. The participants then reproduced the time intervals between the first and the last stimuli by pressing a button. Each participant first conducted experiment 1, then experiment 2, and lastly experiment 3, so that any other possible factors such as rhythm would be the same among participants.

### Data Analysis

The stimulus presentations and data measurements were conducted using psychtoolbox under MATLAB with speakers. First, the time intervals of reproduction were subtracted from those of stimulus presentations. Using these data, to understand the differences and similarities of the time intervals of reproduction among the three types of sessions, Pearson correlation coefficients were calculated among sessions. Second, to understand how the time intervals of reproduction deviated from those of stimulus presentation, in each time interval trial and session, we conducted one sample *t*-test with Bonferroni corrections. Furthermore, the reproduction data were summarized by calculating regression lines to provide separable estimates of uncertainty and bias. Third, we performed a 3 (session: PT, AS, and RS) × 8 (time: 0.8, 1.6, 2.4, 3.2, 4.0, 4.8, 5.6, and 6.4 s trials) repeated-measures ANOVA. The coefficients of variation (standard deviation of estimates divided by the mean reproduced times) were also analyzed to investigate whether there are any differences in variability between conditions. When we detected significant effects, Bonferroni-corrected *post hoc* tests were conducted for further analysis. The statistical significance level was set at *p* = 0.05 for all analyses.

## Results

**FIGURE 2:**
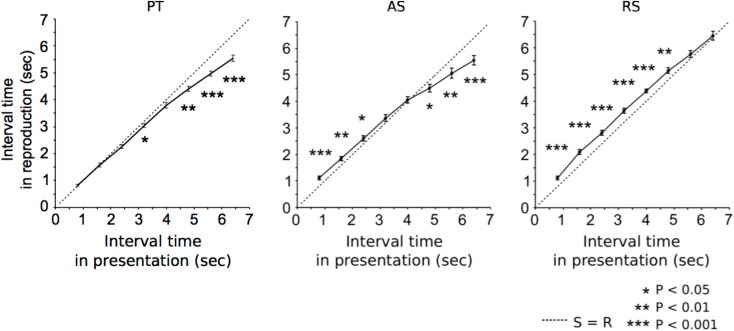
Grand-averaged time intervals (solid lines) that participants reproduced in paired tone (left), accelerando series (middle), and rhythm series sessions (right). At S = R (dashed lines), time intervals between stimuli equals reproductions. The asterisks indicate significant differences from S = R. The error bars indicate the standard error of the mean.

Grand-averaged time intervals of reproduction are shown in **Figure 2**.

### Differences Between Durations of Stimulus and Response and Correlations Between Sessions

In the PT sessions (**Figure 2**, left), the time intervals of reproduction were significantly shorter than those of the presentations in the 3.2 s (*p* = 0.042), 4.8 s (*p* = 0.001), 5.6 s (*p* < 0.001), and 6.4 s trials (*p* < 0.001). In the AS sessions (**Figure 2**, middle), the time intervals of reproduction were significantly longer than those of the presentations in the 0.8 s (*p* < 0.001), 1.6 s (*p* = 0.01), and 2.4 s trials (*p* = 0.047), and shorter than those of the presentations in the 4.8 s (*p* = 0.040), 5.6 s (*p* = 0.008), and 6.4 s trials (*p* < 0.001). In the RS sessions (**Figure 2**, right), the time intervals of reproduction were significantly longer than those of the presentations in the 0.8 s (*p* < 0.001), 1.6 s (*p* < 0.001), 2.4 s (*p* < 0.001), 3.2 s (*p* < 0.001), 4.0 s (*p* < 0.001), and 4.8 s trials (*p* = 0.003). The formula of a regression line in PT, AS, and RS were 0.85x-0.24, 0.95x-0.52, 0.80x-0.77, respectively.

**FIGURE 3:**
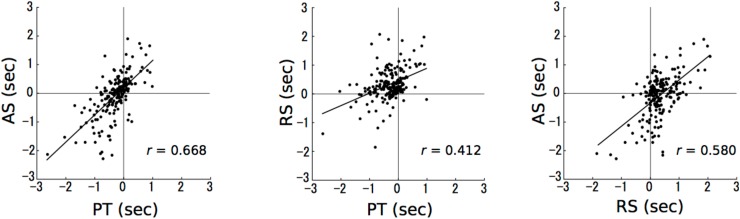
The results of correlation analyses. The numbers in axes represent the differences in time intervals between a stimulus presentation and a reproduction (reproduction – stimulus presentation). The horizontal and vertical axes represent paired tone and accelerando series sessions, respectively (left); paired tone and rhythm series sessions, respectively (middle); and rhythm series and accelerando series sessions, respectively (right). Each data point represents a single participant at a single test duration, so that there are 192 points in each plot [24 (participants) × 8 (durations)].

The PT sessions are moderately (0.4≤| r| <0.7) related to the AS (*r* = 0.668; **Figure 3**, left) and RS sessions (*r* = 0.412; **Figure 3**, middle). The RS sessions are moderately related to the AS sessions (*r* = 0.580; **Figure 3**, right). The correlation coefficients were simply reported without reporting *p*-values, because the data are clustered between durations and violate the independence assumption on which the *p*-values associated with correlations are based.

### ANOVA Results

**FIGURE 4:**
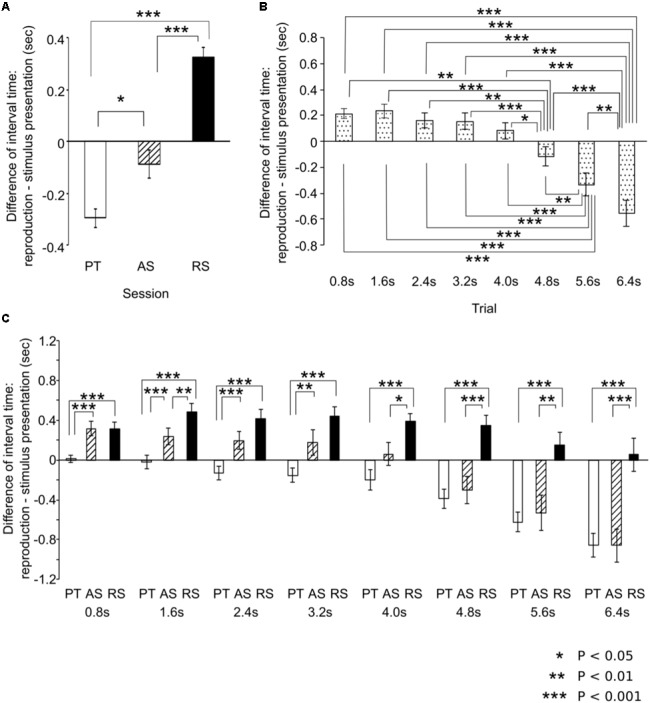
The results of analysis of variances. Based on the main effects of session **(A)** and time intervals **(B)** and the interactions **(C)**, *post hoc* tests were conducted using the Bonferroni correction of significance probability (*p* < 0.05). Until approximately 3 s, the results of the accelerando series sessions are similar to those of the rhythm series sessions whereas from approximately 4 s, they are similar to those of the paired tone sessions. The error bars indicate the standard error of the mean.

The main session effect was significant [*F*(92,46) = 34.20, *p* < 0.001, η^2^ = 0.60; **Figure 4A**) (coefficient of variation based on the mean reproduced times: PT = 0.49, AS = 0.45, RS = 0.46). The time intervals of reproduction in the PT sessions were significantly shorter than those in the AS (*p* = 0.022) and RS sessions (*p* < 0.001). The time intervals of reproduction in the AS sessions were significantly shorter than those in the RS sessions (*p* < 0.001). The main interval effect was significant [*F*(7,161) = 37.76, *p* < 0.001, η^2^ = 0.62; **Figure 4B**) (coefficient of variation based on the mean reproduced times: 0.8 s: 0.33, 2.4 s: 0.24, 3.2 s: 0.16, 4.0 s: 0.13, 4.8 s: 0.14, 5.6 s: 0.14, 6.4 s: 0.15). The time intervals of reproduction in the 6.4 s trial was significantly shorter than those in all types of trials (0.8 s: *p* < 0.001, 1.6 s: *p* < 0.001, 2.4 s: *p* < 0.001, 3.2 s: *p* < 0.001, 4.0 s: *p* < 0.001, 4.8 s: *p* < 0.001, 5.6 s: *p* = 0.012). The time intervals of reproduction in the 5.6 s trial was significantly shorter than those in the 0.8 s (*p* < 0.001), 1.6 s (*p* < 0.001), 2.4 s (*p* < 0.001), 3.2 s (*p* < 0.001), 4.0 s (*p* < 0.001), and 4.8 s (*p* = 0.001) trials. The time intervals of reproduction in the 4.8 s trial was significantly shorter than those in the 0.8 s (*p* = 0.005), 1.6 s (*p* < 0.001), 2.4 s (*p* = 0.001), 3.2 s (*p* < 0.001), and 4.0 s (*p* = 0.019) trials.

The time-session interactions were significant [*F*(14,322) = 6.21, *p* < 0.001, η^2^ = 0.21; **Figure 4C**) (coefficient of variation based on the mean reproduced times: 0.8 s: PT = 0.22, AS = 0.32, RS = 0.30; 1.6 s: PT = 0.22, AS = 0.22, RS = 0.20; 2.4 s: PT = 0.15, AS = 0.17, RS = 0.17; 3.2 s: PT = 0.11, AS = 0.19, RS = 0.13; 4.0 s: PT = 0.14, AS = 0.14, RS = 0.09; 4.8 s: PT = 0.11, AS = 0.15, RS = 0.10; 5.6 s: PT = 0.10, AS = 0.17, RS = 0.11; 6.4 s: PT = 0.10, AS = 0.15, RS = 0.13). In the 0.8, 1.6, 2.4, and 3.2 s trials, the time intervals of reproduction in the PT sessions were significantly shorter than those in the AS sessions (0.8 s: *p* < 0.001, 1.6 s: *p* < 0.001, 2.4 s: *p* < 0.001, 3.2 s: *p* = 0.012) whereas this difference was not detected in the 4.0, 4.8, 5.6, or 6.4 s trials. In contrast, in the 4.0, 4.8, 5.6, and 6.4 s trials, the time intervals of reproduction in the AS sessions were significantly shorter than those in the RS sessions (4.0 s; *p* = 0.040, 4.8 s: *p* < 0.001, 5.6 s: *p* = 0.001, 6.4 s: *p* < 0.001) whereas this difference was not detected in the 0.8, 2.4, or 3.2 s trials. In all types of trials, the time intervals of reproduction in the PT sessions were significantly shorter than those in the RS sessions (*p* < 0.001). In the 1.6 s trial, the time intervals of reproduction in the AS sessions were significantly shorter than those in the RS sessions (*p* = 0.004). In the PT sessions, the time intervals of reproduction in the 6.4 s trial were significantly shorter than those in the 0.8 s (*p* < 0.001), 1.6 s (*p* < 0.001), 2.4 s (*p* < 0.001), 3.2 s (*p* < 0.001), 4.0 s (*p* = 0.003), and 4.8 s (*p* = 0.002) trials. The time intervals of reproduction in the 5.6 s trial were significantly shorter than those in the 0.8 s (*p* < 0.001), 1.6 s (*p* < 0.001), 2.4 s (*p* < 0.001), 3.2 s (*p* = 0.001), 4.0 s (*p* = 0.012), and 4.8 s (*p* = 0.001). The time intervals of reproduction in the 4.8 s trial were significantly shorter than those in the 0.8 s (*p* = 0.004) and 1.6 s (*p* = 0.016) trials. In the AS sessions, the time intervals of reproduction in the 6.4 s trial were significantly shorter than those in the 0.8 s (*p* < 0.001), 1.6 s (*p* < 0.001), 2.4 s (*p* < 0.001), 3.2 s (*p* < 0.001), 4.0 s (*p* < 0.001), 4.8 s (*p* < 0.001), and 5.6 s (*p* = 0.009) trials. The time intervals of reproduction in the 5.6 s trial were significantly shorter than those in the 0.8 s (*p* = 0.001), 1.6 s (*p* < 0.001), 2.4 s (*p* < 0.001), 3.2 s (*p* < 0.001), and 4.0 s (*p* < 0.001) trials. The time intervals of reproduction in the 4.8 s trial were significantly shorter than those in the 0.8 s (*p* = 0.004), 1.6 s (*p* = 0.002), 2.4 s (*p* = 0.002), 3.2 s (*p* < 0.001), and 4.0 s (*p* = 0.002) trials. In the RS sessions, the time intervals of reproduction in the 1.6 s trial were significantly longer than those in the 8.0 s (*p* < 0.024) trials.

## Discussion

### Summary of Previous Findings on Time-Interval Perception and Study Aim

According to previous studies, a time interval of approximately 3 s is the threshold unit of perception in working memory (Poppel, 1997, 2009). A number of behavioral and neurophysiological studies replicated the findings that humans can accurately reproduce time intervals with small temporal variance up to approximately 3 s whereas over 3 s, the time intervals gradually become shorter (Poppel, 1971; Fraisse, 1984; Elbert et al., 1991; Szelag et al., 1996; Wittmann and Poppel, 2000; Ulbrich et al., 2007; Noulhiane et al., 2008). The strategies of time-interval reproduction are, however, influenced by the preceding temporal information that can act as cues for reproduction (Launay et al., 2014). Thus, we hypothesized that the time-interval reproduction depends on the subjective conditions of internalized cues. The present study investigated how temporal processing differed among three conditions: (1) with no internalized cue (paired tone session: PT), (2) with an internalized cue without a beat (accelerando series session: AS), and (3) with an internalized cue with a beat (rhythm series session: RS).

### Time-Interval Perception and Reproduction of 3 s

The results of the PT sessions replicated the previous findings; namely, the participants could accurately reproduce time intervals up to approximately 3 s, and over 3 s, the time intervals in reproduction gradually became shorter (**Figure 2**, left; Poppel, 1971; Fraisse, 1984; Elbert et al., 1991; Szelag et al., 1996; Wittmann and Poppel, 2000; Ulbrich et al., 2007; Noulhiane et al., 2008). The threshold was, however, slightly longer than the 2 to 3 s observed in the studies by Poppel (1997, 2009). There is considerable evidence that the reproduction of a time interval depends significantly on several factors, including stimuli, age, gender, learning abilities, and cognitive function (Szelag, 1997; Szelag et al., 1998, 2002, 2004a,b; Kanabus et al., 2004). The cerebral cortex is a learning machine that can work regardless of attention and domain of learning (Daikoku et al., 2012, 2014, 2015, 2016, 2017a,b,c; Koelsch et al., 2016; Daikoku and Yumoto, 2017; Daikoku, 2018), and that can be developed by cognitive and motor learning throughout life (Merzenich et al., 1996). Szelag et al. (1998) suggested that the prefrontal cortex, which is responsible for the developmental effect, also plays an important role in temporal processing, and is involved in working memory up to around 3 s. Previous studies report that males and older individuals tend to outperform women and younger individuals on perception tests related to temporal processing, respectively (Linn and Petersen, 1985; Szelag et al., 1997, 1998). In the present study, participants had no history of neurological or audiological disorders, and males outnumbered females. The limits of time-interval processing in working memory might be modulated by several factors such as age, gender, learning abilities, and cognitive function.

### Influences of Internalized Temporal Cues in the Brain on Time-Interval Reproduction

The results of the RS sessions showed different tendencies than those of the PT sessions: a shorter presentation of up to 4.8 s and 12 beats induced a consistently longer time interval in reproduction whereas a longer presentation of over 5.6 s and 13 beats induced an accurate time interval in reproduction. According to the previous studies, isochronous sequences such as a beat were perceived as longer than anisochronous ones (Horr and Di Luca, 2015; Horr et al., 2016). Furthermore, Szelag and colleagues reported that lower frequency of a beat leads to longer time intervals, and that a lack of events within a time interval, which leads to a low level of mental and behavioral activities, gives an impression of a slow passage of time, subjectively experienced as boredom (Szelag et al., 1996, 1997; Szelag, 1997). On the other hand, many events within a time interval, which leads to a high level of mental and behavioral activities, give an impression of a quick passage of time. Like these previous studies, the findings of the present study suggest that perceived time varies depending on the precise events occuring within an empty interval.

According to previous studies, temporally adjacent stimuli can be integrated into one percept; this phenomenon also occurs with beat sequences (Szelag et al., 1998). Previous studies also showed that humans prefer isochrony and relative timing based on integer ratios (e.g., 1:2) to non-integer ratios (e.g., 1:2.7) in temporal perception and production (Martin, 1972; Handel and Lawson, 1983; Essens, 1986; Collier and Wright, 1995). We hypothesized that, in the RS sessions, participants might implicitly segregate beat sequences into each perceptive unit based on the accentuations of beats, and integrate and perceive the sequences as a concatenation of units with the same time interval because it is a preferred strategy for memorizing time intervals. The previous study investigated how segmented temporal intervals in tone sequences were recognized (Matthews, 2013). As a result, sequences with equal-sized segments were consistently judged longer than those with accelerating or decelerating structures, in agreement with the findings in the present study. The other studies also suggest that exposure to auditory beat sequences accented every second beat (SOA = 390 ms) for around 5 s leads to the synchronization of ERPs with the beat and the modulation of induced beta-band oscillations (12–30 Hz) in some areas, including the sensorimotor cortices (Fujioka et al., 2010, 2012, 2015; Schaefer et al., 2011). Furthermore, even if the beat sequence disappears, the synchronization of ERPs and the modulation of beta-band oscillations can remain by subjectively imagining the beat. Their results may indicate that, once the neurological synchronization with a beat occurs, the synchronization also participates as a predictor of time intervals by subjectively imagining the beat. The previous studies reported that sensory memory (Palmer and Krumhansl, 1990; Jones et al., 2002) and motor synchronization (Repp, 2007) were facilitated on the downbeat (i.e., accentuation) compared with the upbeat (i.e., the beat preceding the downbeat), suggesting that listeners predict in a top-down manner from the motor to auditory cortices (Jones and Boltz, 1989). The temporal and sensorimotor synchronization of time intervals may be governed by top-down prediction in motor functions based on internalized beats in the brain. In the present study, the shorter presentation up to 4.8 s and 12 beats induced a longer time interval in reproduction whereas the longer presentation over 5.6 s and 13 beats induced an accurate time interval in reproduction. These results may suggest that longer exposure to beat presentation leads to stable internalization and efficiency of sensorimotor processing in perception and reproduction, and that a minimum 5 s and 13 beat presentation is necessary for sensorimotor synchronization and accurate time interval reproduction.

Up to approximately 3 s, the results of the AS sessions were similar to those of the RS sessions whereas over 3 s, they were closer to those of the PT sessions (**Figures 2**, **4C**). Furthermore, the correlation between PT and RS sessions was weaker than the others (**Figure 3**, middle). One possible reason for this is that the time intervals between the adjacent stimuli were made increasingly smaller across each trial. This suggests that, in the AS sessions, the participants could not perceive the sequences as a concatenation of units with the same time interval as they could in the RS session. Another possible reason is that the time-interval processing strategy in the AS sessions was shifted from those in the PT to RS sessions at a time interval of approximately 3 s, based on time intervals between the first two stimuli. In the 2.4 s trial, the time interval between the first two stimuli, which is the longest of all time intervals in an AS session trial, is less than 1 s (0.986 ms). In contrast, in a 3.2 s trial, the time interval between the first two stimuli exceeds 1 s (1.140 ms). The previous studies proposed that the neural basis underlying temporal processing could differ between sub- and supra-seconds. The time-interval processing that takes place within a second is automatic and recruits motor systems in our brain (e.g., the supplementary motor area and the cerebellum) (Sakai et al., 1999; Schubotz and von Cramon, 2001; Coull et al., 2004; Pastor et al., 2004). In contrast, the time-interval processing that takes longer than a second depends on working memory and is more connected to the right prefrontal and parietal cortical areas (Madison, 2001; Lewis and Miall, 2003; Miyake et al., 2004). The neural basis underlying the reproduction of time intervals without a beat may depend on the length of the time interval between adjacent stimuli in the sequences. It cannot be, however, excluded the possibility that the differences of performance between the three conditions are the result of the particular order in which participants were presented and representing some combination of practice or fatigue effects. Future study is necessary to investigate under many types of conditions.

### Sensorimotor System in Temporal Processing

As a general tendency shared among the three sessions, the reproduction error gradually became more negative as the reproduced interval increased (**Figures 2**, **4B**). The tendencies were correlated with each other (**Figure 3**). This finding has also been detected in previous studies (Poppel, 1971; Fraisse, 1984; Elbert et al., 1991; Szelag et al., 1996; Szelag, 1997; Szelag et al., 1998; Wittmann and Poppel, 2000; Ulbrich et al., 2007; Noulhiane et al., 2008). Thus, it may be a universal phenomenon of time-interval processing in the human brain, regardless of internalized cues. According to the Paillard–Fraisse hypothesis (Paillard, 1949; Fraisse, 1980), the brain can synchronize the motor activities of tapping with auditory stimuli by superimposing the auditory code and the tactile/kinesthetic code in time. The tap and auditory stimulus coincide exactly, however, because auditory processing times differ from tactile processing times. It was indicated that it takes more time for sensory information to travel from the motor cortex to the brain than from the auditory cortex to the brain (Aschersleben and Prinz, 1995). Thus, sensorimotor synchronization has inevitable time lags because the neural circuits and distance differ between the motor cortex and the brain and the auditory cortex and the brain. As a result, when humans try to synchronize tapping with a regular sequence of auditory stimulus, the tapping consistently precedes the sensory stimuli (Dunlap, 1910; Peters, 1989; Mates et al., 1992; Aschersleben and Prinz, 1995). According to previous studies, exposure to auditory beat sequences leads to the synchronization of ERPs with the beat and the modulation of induced beta oscillations in the sensorimotor cortices (Fujioka et al., 2010, 2012, 2015; Schaefer et al., 2011). Furthermore, even if the beat sequence disappears, the synchronization of ERPs and the modulation of beta-band oscillations can be retained by subjectively imagining the beat. Thus, it is possible that once neurological synchronization with a beat occurs, the synchronization can also function as a predictor of time intervals by subjectively imagining the beat. The previous studies also suggest that the motor cortex contributes to prediction about when and what auditory stimuli are presented, and that their functions can be reflected in beta oscillations and ERP activities (Jones and Boltz, 1989; van Wassenhove et al., 2005; Mottonen et al., 2013, 2014). The temporal and sensorimotor synchronization of time intervals may be governed by top-down prediction in motor functions (Jones and Boltz, 1989). Thus, tapping may also precede imagery sensory stimuli, because the neural circuits and distance differ between the motor cortex and the brain and the auditory cortex and the brain (Dunlap, 1910; Peters, 1989; Mates et al., 1992; Aschersleben and Prinz, 1995). Because of a small set of subjects with a low number of trials in each condition, and only three conditions in the present study, however, it is difficult to conclude the findings are general phenomena in humans. To extensively understand cognitive function of time interval, future study is necessary to examine how humans reproduce time intervals when they received feedback, when the accelerando session was reversed so that stimuli decreased in speed throughout the interval (i.e., ritardando).

In sum, the present study replicated the findings of a number of previous studies: in PT sessions, participants accurately reproduced time intervals up to approximately 3 s whereas over 3 s, the time intervals in reproduction became shorter. In contrast, in RS sessions, the longer presentation of over 5.6 s and 13 beats induced accurate time intervals in reproduction. This may suggest that longer exposure to beat presentation leads to stable internalization and efficiency of sensorimotor processing in perception and reproduction. In AS sessions, up to approximately 3 s, the results were similar to those of RS sessions whereas over 3 s, they shifted to be more similar to those of PT sessions. Based on the time intervals between the first two stimuli, the strategies of time-interval reproduction in AS sessions may shift from RS to PT sessions. The neural basis underlying the reproduction of time intervals without a beat may depend on the length of the time interval between adjacent stimuli in the sequences. The neural and behavioral performance on time-interval reproduction could be a useful clinical biomarker of impairments for rehabilitation and for the early diagnosis of risk factors in developmental disorders. Furthermore, time processing can benefit persons with motor and other impairments such as stuttering (Toyomura et al., 2015), Parkinson’s disease, stroke (Thaut et al., 2015), dyslexia (Przybylski et al., 2013), and autism (Szelag et al., 2014). Further research is needed to understand how time-interval reproduction differs among individuals and among specific impairments, and to determine the essential and universal phenomena of temporal processing in humans.

## Conclusion

The present study detected four types of results. First, our findings were in accordance with those of a number of previous studies: in PT sessions, participants accurately reproduced time intervals up to approximately 3 s whereas over 3 s, the time intervals in reproduction became shorter. Second, the present study detected the general phenomenon of temporal processing in humans, namely, that the time intervals in reproduction gradually become shorter, regardless of internalized cues, in the human brain. Third, in RS sessions, a longer presentation over 5.6 s and 13 beats induced accurate time intervals in reproduction. This may suggest that longer exposure to beat presentation leads to stable internalization and efficiency of sensorimotor processing in perception and reproduction. Fourth, in AS sessions, up to approximately 3 s, the results were similar to those of RS sessions whereas over 3 s, they shifted to become similar to those of PT sessions. Based on the time intervals between the first two stimuli, the strategies of time-interval reproduction in AS sessions may shift from RS to PT sessions. The neural basis underlying the reproduction of time intervals without a beat may depend on the length of the time interval between adjacent stimuli in the sequences. Previous studies suggest that the neural and behavioral performance on time-interval reproduction could be a useful clinical biomarker of impairments for rehabilitation and for the early diagnosis of risk factors in developmental disorders. Furthermore, time processing could benefit persons with motor and other impairments, such as stuttering, Parkinson’s disease, stroke, dyslexia, and autism. Further research is needed to understand how time-interval reproduction differs among individuals and among specific impairments, and to determine the essential and universal phenomena of temporal processing in humans.

## Ethics Statement

All procedures performed in studies involving human participants were in accordance with the ethical standards of the national research committee and with the 1964 Helsinki declaration and its later amendments or comparable ethical standards. Informed consent was obtained from all individual participants included in the study.

## Author Contributions

TD devised the paradigm and YT recruited subjects and did experiment. TD analyzed the data and all authors discussed about the results. TD first wrote the draft of article, then all authors refined it.

## Conflict of Interest Statement

The authors declare that the research was conducted in the absence of any commercial or financial relationships that could be construed as a potential conflict of interest.
